# Supplementary material for: Impacts of climate change on diarrhoeal disease hospitalisations: How does the global warming targets of 1.5–2°C affect Dhaka, Bangladesh?
Source: PLoS Negl Trop Dis. 2024 Sep 26;18(9):e0012139. doi: 10.1371/journal.pntd.0012139 (PMC11426472; doi:10.1371/journal.pntd.0012139)
Supplement: S2 File — (DOCX) [file pntd.0012139.s002.docx]

**S2 File. Conceptual framework for considering factors as potential confounders/covariates/effect modifiers in the models investigating the relationship between temperature (explanatory/independent variable) and diarrhoea hospitalisation (response/dependent variable)**

| **Factor** | **Level** | **Vary on a daily basis** | **Effect on exposure** | **Effect on outcome** | **Confounder/**  **covariate/**  **effect modifier** |
| --- | --- | --- | --- | --- | --- |
| Relative humidity | Population | Yes | Yes | Affect the replication of bacterial and protozoal diarrhoeal disease pathogens and survival of enteroviruses [1-4]. | Confounder |
| Heavy rainfall | Population | Yes | Yes | Heavy rainfall increases the occurrence of diarrhoeal disease by increasing contamination of drinking water. Heavy rainfall increases host susceptibility to infection by causing malnutrition through crop/livestock destruction and reduced agricultural yield [5]. | Confounder |
| Rainfall | Population | Yes | Yes | Excess/scanty rainfall can cause floods/droughts and lead to microbial contamination of drinking water causing diarrhoea [1-4, 6, 7] | Confounder |
| Seasonality/Long-term trend | Population | Yes | Possible (seasonal scale) | Diarrhoea is seasonal. Cholera has a dual peak in the year [1-4, 6]. | Confounder/covariate |
| Day of the week vs weekend | Population | Yes | May be | Access to healthcare and diarrhoea hospitalisation rates may vary between weekdays and weekends [3, 4, 6-10]. | Covariate |
| Age | Individual | No | None | Young age under 5 and adults over 70 years are more susceptible to diarrhoea mortality [8, 11]. | Effect modifier |
| Sex | Individual | No | None | Males have higher incidence than girls. However, males are likely to access healthcare more than females. Women may be more vulnerable due to caregiver responsibility and low access to healthcare [8, 11-13]. | Effect modifier |
| Population density | Population | No | None | High population density is associated with more diarrhoea [8]. | Effect modifier |
| Access to improved water supply | Population | No | None | Poor access to improved drinking water supply leads to excess diarrhoea [8, 14-17]. | Effect modifier |
| Sanitation coverage | Population | No | None | Poor sanitation coverage leads to excess diarrhoea [15, 16, 18]. | Effect modifier |

*Investigating confounders/covariates*

In statistics, a confounder (also known as a confounding variable, confounding factor, or lurking variable) is a variable that influences both the dependent variable and independent variable, causing a spurious association. Unlike normal regression analysis, in a time series analysis, the main unit of analysis is the day and not the individual person. This is an important point when considering what the potential confounders might be in the analysis [19]. In general epidemiology, common confounders include age, gender and literacy level but these confounders do not apply to the time series regression analysis because at the population level, the distribution of these factors are unlikely to change on a day-to-day basis and cannot be associated with fluctuations in independent variables i.e. the environmental exposures such as ambient temperature [19, 20]. Potential time-varying confounders such as relative humidity, cumulative rainfall and atmospheric pressure, which can change on a daily basis and may affect both the outcome and exposure of interest, was incorporated into the model to control for confounders. The procedure was repeated with the different meteorological variables. Only those associations for which meaningful correlations were found, were included in the final model allowing for non-linearity where expected [19]. We also incorporated covariates in the model. A covariate was a variable that was related to the dependent variable but was not the main focus of the study. These were included in the analysis to account for their potential influence on the dependent variable. Covariates were used to increase the precision and accuracy of estimates by reducing the error variance.

*Investigating effect modification*

In randomised controlled trials and regression models using individual data, modification of the effect of association by a third variable (i.e. effect modifier) can be examined using various approaches. Typically, factors of interest are tested and tried in models to assess whether these explain the variability in the outcome. Only if explanatory of the outcome of interest, a factor is tested if it affects the relationship of interest. The usual statistical approach for evaluating potential effect modifiers is a test for statistical interaction [21, 22]. Frequently, interaction terms are included into the model and if found statistically significant (P-value <0.05), there is evidence that a synergy effect exists between these variables. However, in time series regression models using grouped data, such statistical tests of interactions cannot be applied [19, 20]. As a result, potential effect modifiers were identified a priori through review of the existing literature. Finally, sub-group analysis was conducted, and stratum-specific measures of association reported to highlight effect modification. Table 1S provides the conceptual framework for considering factors as potential confounders/covariates/effect modifiers.

**References**

1. Feldacker, C.B. *Environmental influences on cholera outbreaks in Bangladesh and Vietnam: implications for prevention and prediction*. in *APHA 135th Meeting and Expo*. 2007. Washington, DC.

2. Huq, A., R. Sack, and A. Nizam, *Critical factors influencing the occurrence of Vibrio cholerae in the environment of Bangladesh.* Appl Environ Microbiol, 2005. **71**: p. 4645-4654.

3. Levy, K., S.M. Smith, and E.J. Carlton, *Climate Change Impacts on Waterborne Diseases: Moving Toward Designing Interventions.* Curr Environ Health Rep, 2018. **5**(2): p. 272-282.

4. Levy, K., et al., *Untangling the Impacts of Climate Change on Waterborne Diseases: a Systematic Review of Relationships between Diarrheal Diseases and Temperature, Rainfall, Flooding, and Drought.* Environ Sci Technol, 2016. **50**(10): p. 4905-22.

5. Carlton, E.J., et al., *Heavy rainfall events and diarrhea incidence: the role of social and environmental factors.* Am J Epidemiol, 2014. **179**(3): p. 344-52.

6. Hashizume, M., et al., *Association between climate variability and hospital visits for non-cholera diarrhoea in Bangladesh: effects and vulnerable groups.* International Journal of Epidemiology, 2007. **36**: p. 1030–1037.

7. Hashizume, M., et al., *Rotavirus infections and climate variability in Dhaka, Bangladesh: a time-series analysis.* Epidemiol Infect, 2008. **136**: p. 1281-1289.

8. Colombara, D.V., et al., *Risk factors for diarrhea hospitalization in Bangladesh, 2000-2008: a case-case study of cholera and shigellosis.* BMC Infect Dis, 2014. **14**: p. 440.

9. Hashizume, M., et al., *The effect of temperature on mortality in rural Bangladesh--a population-based time-series study.* Int J Epidemiol, 2009. **38**(6): p. 1689-97.

10. Imai, C., et al., *Time series regression model for infectious disease and weather.* Environ Res, 2015. **142**: p. 319-27.

11. Jarman, A.F., et al., *Sex and Gender Differences in Acute Pediatric Diarrhea: A Secondary Analysis of the Dhaka Study.* J Epidemiol Glob Health, 2018. **8**(1-2): p. 42-47.

12. Chen, L., E. Huq, and S. D'Souza, *Sex bias in the Family Allocation of Food and Health Care in Rural Bangladesh* Population and Development Review, 1981. **7**: p. 55-70.

13. WHO, *Addressing sex and gender in epidemic-prone infectious diseases*. 2007, World Health Organization Geneva.

14. Clasen, T., S. Nadakatti, and S. Menon, *Microbiological performance of a water treatment unit designed for household use in developing countries.* Trop Med Int Health, 2006. **11**(9): p. 1399-405.

15. Clasen, T.F., *Household water treatment and the millennium development goals: keeping the focus on health.* Environ Sci Technol, 2010. **44**(19): p. 7357-60.

16. Pickering, A.J., et al., *Hands, water, and health: fecal contamination in Tanzanian communities with improved, non-networked water supplies.* Environ Sci Technol, 2010. **44**(9): p. 3267-72.

17. Shaheed, A., et al., *Water quality risks of 'improved' water sources: evidence from Cambodia.* Trop Med Int Health, 2014. **19**(2): p. 186-94.

18. Fewtrell, L., et al., *Water, sanitation, and hygiene interventions to reduce diarrhoea in less developed countries: a systematic review and meta-analysis.* Lancet Infect Dis, 2005. **5**(1): p. 42-52.

19. Bhaskaran, K., et al., *Time series regression studies in environmental epidemiology.* Int J Epidemiol, 2013. **42**(4): p. 1187-95.

20. Shumway, R.H. and D.S. Stoffer, eds. *Time Series Analysis and Its Applications* Fourth ed. Springer Texts in Statistics 2017, Springer International Publishing AG: Switzerland

21. Altman, D.G. and J.M. Bland, *Interaction revisited: the difference between two estimates.* BMJ, 2003. **326**(7382): p. 219.

22. Christensen, R., M.J.L. Bours, and S.M. Nielsen, *Effect Modifiers and Statistical Tests for Interaction in Randomized Trials.* J Clin Epidemiol, 2021. **134**: p. 174-177.
