# Supplementary material for: Impacts of climate change on diarrhoeal disease hospitalisations: How does the global warming targets of 1.5–2°C affect Dhaka, Bangladesh?
Source: PLoS Negl Trop Dis. 2024 Sep 26;18(9):e0012139. doi: 10.1371/journal.pntd.0012139 (PMC11426472; doi:10.1371/journal.pntd.0012139)
Supplement: S3 File — (DOCX) [file pntd.0012139.s003.docx]

**S3 File. Sensitivity analyses and model diagnostics.**

Sensitivity analysis was conducted by varying the amount of control for long-term trend and seasonality. Initially, the analyses were repeated using 1-14 degrees of freedom per year to control for long-term trend and seasonality to check the robustness of the results.

For the sensitivity analysis, the model took the following form:

*Y_t_ ~ Negative Binomial (µt, θ)*


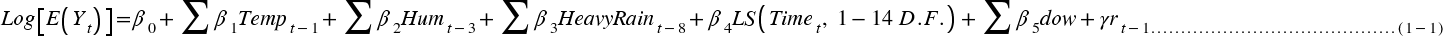


To allow for seasonality and long-terms trends in the data, natural cubic spline function of time was fitted instead of linear spline. The analyses were repeated using 3–7 degrees of freedom per year for calculating the number of knots in order to estimate whether the results were sensitive to the levels of control for the long-term trend and seasonal patterns.

For the sensitivity analysis, the model took the following form:

*Y_t_ ~ Negative Binomial (µt, θ)*


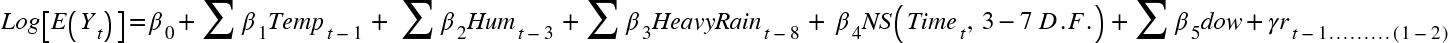


Further analysis was conducted by including the population of Dhaka into the model as an offset. The offset provides a way to insert a constant term directly into the linear predictor without that term’s being estimated. The offset can influence the algorithm directly before and after regression estimation. The offset was first introduced by John Nelder and can be utilised for modelling rate data. Epidemiologists frequently interpret offset as an exposure where the number of underlying population are regarded as the people who are exposed to a disease and the incidence, or the number of people who suffer the outcome enter as the response. Including offset in this way allows the negative binomial model to be parameterized as a rate, as modelling incidence over exposure. The mean and variance function remains identical and there is no variation in how the count enters into the group even after including the offset. However, the offset needs to enter the canonical link negative binomial model as the natural logarithm of the variable as the offset must be put into the same metric as the linear predictor [1].

After including the offset, the model took the following form:


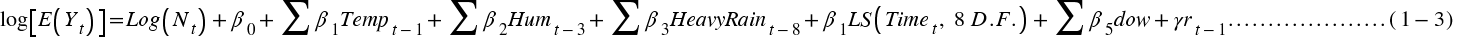


where, Log(*N_t_*) is the model offset (i.e. the population of Dhaka).

To explore to what extent the changes in the sample size affected the results, the models were re-run using the actual number of diarrheal disease patients enrolled into the surveillance system (DDSS) instead of the total estimated number of patients with diarrheal diseases in the Dhaka Hospital during the study period.


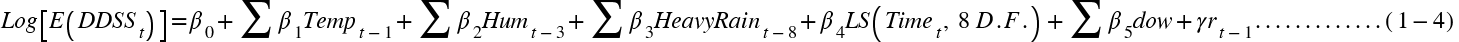


Furthermore, two separate time-series negative binomial models were generated to explore the relationship between daily diarrhoea hospitalisation and daily mean temperature, relative humidity and heavy rainfall and WASH interventions including access to safe water and improved sanitation as indicator variables using the following models:


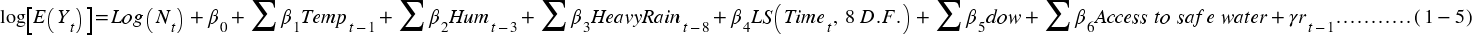


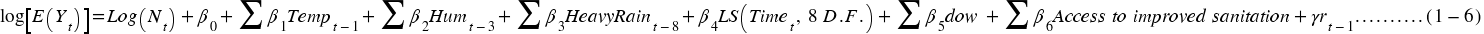


**Findings**

The results of the sensitivity analysis are shown in Table A and Table B in S3 File. All results are comparable to the primary analysis. The models with the lowest BIC values were considered as the best models.

**Table A. Incidence rate ration (IRR) of diarrhoea hospitalisation in all ages and under-5 children associated with 1°C increase in temperature in Dhaka Bangladesh**

|  | **All ages** | | | **Under-5 children** | | |
| --- | --- | --- | --- | --- | --- | --- |
|  | **IRR (95% CI)** | **BIC** | **Dispersion** | **IRR (95% CI)** | **BIC** | **Dispersion** |
| D.F. | Using Equation 1-1 | | | |  |  |
| 1 | 1.0107 (1.0097 – 1.0117) | 11657.3 | 1.0417 | 1.0191 (1.0181 – 1.0273) | 12380.5 | 1.0598 |
| 2 | 1.0115 (1.0104 – 1.0126) | 11661.9 | 1.0375 | 1.0251 (1.0111 – 1.0303) | 12364.9 | 1.0586 |
| 3 | 1.0141 (1.0123 – 1.0155) | 11655.7 | 1.0417 | 1.0291 (1.0181 – 1.0373) | 12386.1 | 1.0571 |
| 4 | 1.0159 (1.0140 – 1.0178) | 11643.5 | 1.0460 | 1.0321 (1.0256 – 1.0370) | 12345.2 | 1.0543 |
| 5 | 1.0190 (1.0169 – 1.0212) | 11659.6 | 1.0495 | 1.0351 (1.0312 – 1.0393) | 12302.9 | 1.0532 |
| 6 | 1.0221 (1.0198 – 1.0244) | 11632.8 | 1.0532 | 1.0372 (1.0351 – 1.0404) | 12299.6 | 1.0501 |
| 7 | 1.0322 (1.0221 – 1.0268) | 11609.9 | 1.0568 | 1.0381 (1.0371 – 1.0451) | 12293.3 | 1.0497 |
| 8* | 1.0335 (1.0304 – 1.0367) | 11586.7 | 1.0143 | 1.0391 (1.0381 – 1.0473) | 12281.9 | 1.0374 |
| 9 | 1.0328 (1.0224 – 1.0371) | 11597.2 | 1.0657 | 1.0371 (1.0356 – 1.0423) | 12296.2 | 1.0489 |
| 10 | 1.0256 (1.0232 – 1.0280) | 11602.9 | 1.0696 | 1.0322 (1.0301 – 1.0417) | 12301.1 | 1.0494 |
| 11 | 1.0261 (1.0238 – 1.0285) | 11616.0 | 1.0729 | 1.0341 (1.0322 – 1.0393) | 12322.4 | 1.0499 |
| 12 | 1.0263 (1.0239 – 1.0287) | 11623.9 | 1.0769 | 1.0297 (1.0281 – 1.0352) | 12345.3 | 1.0517 |
| 13 | 1.0264 (1.0240 – 1.0288) | 11625.3 | 1.0810 | 1.0271 (1.0255 – 1.0324) | 12357.5 | 1.0523 |
| 14 | 1.0265 (1.0241 – 1.0289) | 11660.8 | 1.0837 | 1.0252 (1.0181 – 1.0313) | 12369.2 | 1.0539 |
| D.F. | Using Equation 1-2 |  |  |  |  |  |
| 3 | 1.0299 (1.0267 – 1.0332) | 11675.3 | 1.0310 | 1.0314 (1.0238 – 1.0332) | 12392.6 | 1.0586 |
| 4 | 1.0300 (1.0267 – 1.0332) | 11667.3 | 1.0306 | 1.0321 (1.0244 – 1.0341) | 12399.4 | 1.0579 |
| 5 | 1.0300 (1.0268 – 1.0331) | 11637.1 | 1.0317 | 1.0332 (1.0298 – 1.0355) | 12386.7 | 1.0554 |
| 6 | 1.0300 (1.0268 – 1.0332) | 11635.5 | 1.0320 | 1.0344 (1.0328 – 1.0363) | 12370.1 | 1.0485 |
| 7 | 1.0300 (1.0268 – 1.0331) | 11637.0 | 1.0320 | 1.0336 (1.0317 – 1.0363) | 12379.2 | 1.0493 |
| D.F | Using Equation 1-3 | | | | | |
| 8 | 1.0330 (1.0220 – 1.0530) | 12540.8 | 0.9325 | 1.0341 (1.0298 – 1.0391) | 12295.8 | 1.0392 |
| D.F. | Using Equation 1-4 | | | | | |
| 8 | 1.0322 (1.0254 – 1.0391) | 12667.2 | 0.9341 | 1.0370 (1.0311 – 1.0402) | 12298.2 | 1.0401 |
| D.F. | Using Equation 1-5 |  |  |  |  |  |
| 8 | 1.0334 (1.0154 – 1.0517) | 11602.6 | 1.0575 | 1.0385 (1.0335 – 1.0453) | 12300.2 | 1.0572 |
| D.F. | Using Equation 1-6 |  |  |  |  |  |
| 8 | 1.0334 (1.0154 – 1.0517) | 11605.2 | 1.0577 | 1.0384 (1.0341 – 1.0452) | 12305.1 | 1.0578 |

*Represents the models with the lowest BIC values.

|  | **Males** | | | **Females** | | |
| --- | --- | --- | --- | --- | --- | --- |
|  | **Incidence Rate Ratio IRR (95% CI)** | **BIC** | **Dispersion** | **Incidence Rate Ratio IRR (95% CI)** | **BIC** | **Dispersion** |
| D.F. | Equation 1-1 | | | | | |
| 1 | 1.0151 (1.0114 – 1.0232) | 12423.4 | 1.0606 | 1.0142 (1.0111 - 1.0239) | 12396.5 | 1.0705 |
| 2 | 1.0193 (1.0174 – 1.0331) | 12410.5 | 1.0625 | 1.0185 (1.0158 - 1.0299) | 12391.6 | 1.0701 |
| 3 | 1.0224 (1.0202 – 1.0353) | 12406.1 | 1.0638 | 1.0201 (1.0187 - 1.0302) | 12382.7 | 1.0698 |
| 4 | 1.0273 (1.0219 – 1.0362) | 12398.8 | 1.0648 | 1.0243 (1.0208 - 1.0341) | 12378.2 | 1.0652 |
| 5 | 1.0315 (1.0274 – 1.0372) | 12391.1 | 1.0633 | 1.0289 (1.0257 - 1.0361) | 12354.2 | 1.0643 |
| 6 | 1.0329 (1.0281 – 1.0384) | 12382.9 | 1.0612 | 1.0302 (1.0278 - 1.0374) | 12334.8 | 1.0638 |
| 7 | 1.0344 (1.0299 – 1.0406) | 12381.7 | 1.0598 | 1.0331 (1.0287 - 1.0401) | 12312.9 | 1.0620 |
| 8* | 1.0353 (1.0274 – 1.0432) | 12326.3 | 1.0505 | 1.0344 (1.0228 - 1.0461) | 12285.2 | 1.0608 |
| 9 | 1.0343 (1.0262 – 1.0414) | 12346.2 | 1.0545 | 1.0340 (1.0221 - 1.0459) | 12355.4 | 1.0622 |
| 10 | 1.0322 (1.0251 – 1.0409) | 12359.1 | 1.0566 | 1.0333 (1.0219 - 1.0451) | 12367.6 | 1.0635 |
| 11 | 1.0310 (1.0246 – 1.0399) | 12368.6 | 1.0578 | 1.0329 (1.0208 - 1.0444) | 12382.3 | 1.0648 |
| 12 | 1.0278 (1.0225 – 1.0363) | 12374.2 | 1.0589 | 1.0321 (1.0202 - 1.0437) | 12388.5 | 1.0657 |
| 13 | 1.0256 (1.0212 – 1.0351) | 12365.5 | 1.0592 | 1.0315 (1.0198 - 1.0389) | 12395.9 | 1.0662 |
| 14 | 1.0223 (1.0201 – 1.0342) | 12352.8 | 1.0593 | 1.0309 (1.0189 - 1.0342) | 12398.2 | 1.0675 |
| D.F. | Equation 1-2 | | | | | |
| 3 | 1.0308 (1.0294 – 1.0362) | 12348.5 | 1.0595 | 1.0289 (1.0201 - 1.0445) | 12312.2 | 1.0698 |
| 4 | 1.0312 (1.0299 – 1.0371) | 12356.1 | 1.0592 | 1.0304 (1.0212 - 1.0451) | 12305.8 | 1.0685 |
| 5 | 1.0321 (1.0305 – 1.0384) | 12364.3 | 1.0546 | 1.0313 (1.0223 - 1.0455) | 12295.6 | 1.0674 |
| 6 | 1.0333 (1.0311 – 1.0392) | 12332.2 | 1.0515 | 1.0322 (1.0228 - 1.0461) | 12288.6 | 1.0653 |
| 7 | 1.0324 (1.0305 – 1.0388) | 12352.6 | 1.0539 | 1.0311 (1.0218 - 1.0463) | 12293.1 | 1.0666 |
| D.F | Equation 1-3 | | | | | |
| 8 | 1.0335 (1.0206 – 1.0501) | 12355.5 | 1.0523 | 1.0325 (1.0208 - 1.0499) | 12292.1 | 1.0625 |
| D.F. | Equation 1-4 | | | | | |
| 8 | 1.0334 (1.0224 – 1.0504) | 12361.3 | 1.0542 | 1.0321 (1.0219 - 1.0501) | 12305.5 | 1.0623 |
| D.F. | Equation 1-5 | | | | | |
| 8 | 1.0335 (1.0167 – 1.0531) | 12388.8 | 1.0536 | 1.0326 (1.0171 – 1.0532) | 12352.6 | 1.0632 |
| D.F. | Equation 1-6 | | | | | |
| 8 | 1.0333 (1.0168 – 1.0532) | 12379.1 | 1.0532 | 1.0327 (1.0188 – 1.0556) | 12358.2 | 1.0635 |

**Table B. Incidence rate ration (IRR) of diarrhoea hospitalisation in males and females associated with 1°C increase in temperature in Dhaka Bangladesh**

**Fig A. Partial autocorrelation function plot of deviance residuals of the regression models for all ages (upper left), under-5 children (upper right), males (lower left) and females (lower right) adjusted for autocorrelation**

**
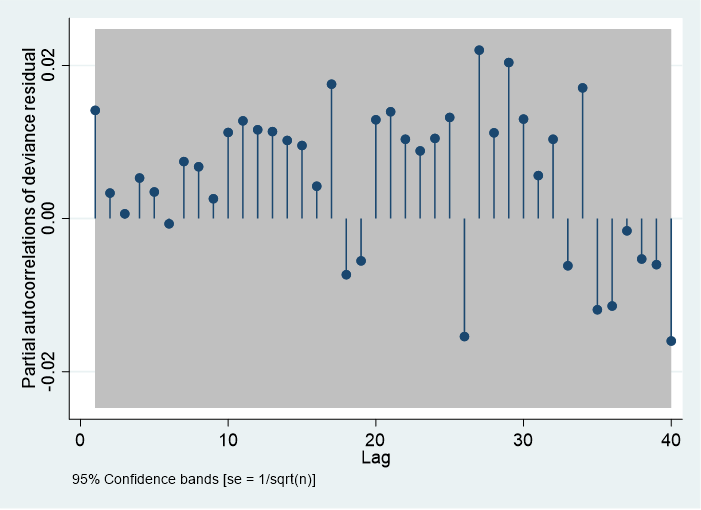

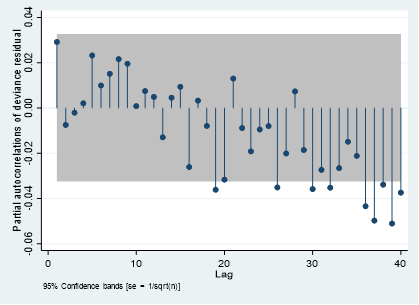

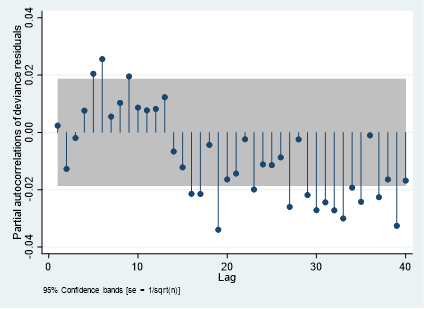

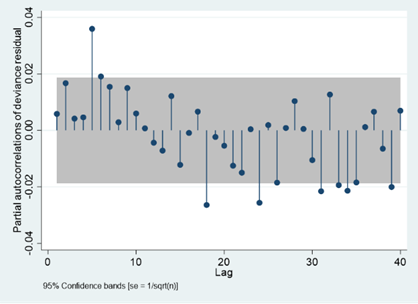
**

**References**

1. Hardin, J.W. and J.M. Hilbe, eds. Generalized Linear Models and Extensions 2nd ed. 2007, Stata Press: Texas 77845.

2. Hardin JW, Hilbe JM, editors. Generalized Linear Models and Extensions. Fourth ed. Texas: StataCorp LP; 2018.

3. Chandler RE, Scottt EM, editors. Statistical Methods for Trend Detection and Analysis in the Environmental Sciences. First ed. United Kingdom John Wiley & Sons, Ltd; 2011.

4. Imai C, Armstrong B, Chalabi Z, Mangtani P, Hashizume M. Time series regression model for infectious disease and weather. Environ Res. 2015;142:319-27.

5. Becketti S, editor. Introduction to Time Series Using Stata. Revised ed. College Station, Texas Stata Press 2020.

6. Bhaskaran K, Gasparrini A, Hajat S, Smeeth L, Armstrong B. Time series regression studies in environmental epidemiology. Int J Epidemiol. 2013;42(4):1187-95.
