# Supplementary material for: Impacts of climate change on diarrhoeal disease hospitalisations: How does the global warming targets of 1.5–2°C affect Dhaka, Bangladesh?
Source: PLoS Negl Trop Dis. 2024 Sep 26;18(9):e0012139. doi: 10.1371/journal.pntd.0012139 (PMC11426472; doi:10.1371/journal.pntd.0012139)
Supplement: S4 File — (DOCX) [file pntd.0012139.s004.docx]

**S4 File. Descriptions/definitions of climate terms and jargon**

- **GCMs:** A global climate model (GCM) is a complex mathematical representation of the major climate system components (atmosphere, land surface, ocean, and sea ice), and their interactions. Earth's energy balance between the four components is the key to long-term climate prediction [1].
- **RCMs:** Regional climate models (RCMs) are numerical models that simulate the climate of geographic regions typically covering a few thousand square kilometres to a continent. Most regional climate models include models that describe the atmosphere and the underlying land surface, but a few also include models of ocean and sea ice and atmospheric aerosols and chemistry [2].
- **Representative Concentration Pathways (RCPs):** RCPs are a method for capturing the assumptions about the economic, social and physical changes to the environment that will influence climate change within a set of scenarios. The conditions of each scenario are used in the process of modelling possible future climate evolution [3].
- **Multi-model ensemble (MME):** A method for combining different, independent climate models [4].
- **Coupled Model Intercomparison Project Phase (CMIP)**: CMIP is a project of the World Climate Research Programme (WCRP) providing climate projections to understand past, present and future climate changes [5].
- **Negative binomial:** The negative binomial (NB) model is similar to the Poisson model but incorporates an additional term to account for the excess variance in the outcome variable [6, 7].
- **Overdispersion:** Overdispersion means that the variance of the response is greater than what's assumed by the model [6, 7].
- **Autocorrelation:** Autocorrelation refers to the degree of correlation of the same variables between two successive time intervals. It measures how the lagged version of the value of a variable is related to the original version of it in a time series. Autocorrelation, as a statistical concept, is also known as serial correlation [6, 7].
- **WASH (Water, Sanitation and Hygiene):** WASH is a sector in development cooperation or within local governments that deals with provision of different water, sanitation, and hygiene services to people.
- **Bayesian information criterion (BIC):** The BIC is a criterion for model selection among a finite set of models; models with lower BIC are generally preferred. It is based, in part, on the likelihood function and it is closely related to the Akaike information criterion (AIC). When fitting models, it is possible to increase the maximum likelihood by adding parameters, but doing so may result in overfitting. Both BIC and AIC attempt to resolve this problem by introducing a penalty term for the number of parameters in the model; the penalty term is larger in BIC than in AIC for sample sizes greater than 7 [6, 7].
- **Generalized estimating equation (GEE):** The GEE is used to estimate the parameters of a generalized linear model with a possible unmeasured correlation between observations from different timepoints. Regression beta coefficient estimates from the Liang-Zeger GEE are consistent, unbiased, and asymptotically normal even when the working correlation is misspecified, under mild regularity conditions. GEE is higher in efficiency than generalized linear iterative model (GLIM) in the presence of high autocorrelation[6, 7].

**References**

- 1. Pierce DW, Barnett TP, Santer BD, Gleckler PJ. Selecting global climate models for regional climate change studies. Proc Natl Acad Sci U S A. 2009;106(21):8441-6. Epub 20090513. doi: 10.1073/pnas.0900094106. PubMed PMID: 19439652; PubMed Central PMCID: PMCPMC2689003.
- 2. Giorgi F. Thirty Years of Regional Climate Modeling: Where Are We and Where Are We Going next? Journal of Geophysical Research 2019;124(11). doi: <https://doi.org/10.1029/2018JD030094>.
- 3. Vuuren DPv, Edmonds J, Kainuma M, Riahi K, Thomson A, Hibbard K, et al. The representative concentration pathways: an overview. Climate Change 2011;109:5-31. doi: 10.1007/s10584-011-0148-z.
- 4. Jose DM, Vincent AM, Dwarakish GS. Improving multiple model ensemble predictions of daily precipitation and temperature through machine learning techniques. Sci Rep. 2022;12(1):4678. Epub 20220318. doi: 10.1038/s41598-022-08786-w. PubMed PMID: 35304552; PubMed Central PMCID: PMCPMC8933560.
- 5. Touzé-Peiffer L, Barberousse A, Treut HL. The Coupled Model Intercomparison Project: History, uses, and structural effects on climate research. WIREs Clim Change. 2020;11(e648). doi: 10.1002/wcc.648.
- 6. Hardin JW, Hilbe JM, editors. Generalized Linear Models and Extensions 2nd ed. Texas 77845: Stata Press; 2007.
- 7. Hardin JW, Hilbe JM, editors. Generalized Linear Models and Extensions. Fourth ed. Texas: StataCorp LP; 2018.
